# Cancer Stem Cell-Related Gene Periostin: A Novel Prognostic Marker for Breast Cancer

**DOI:** 10.1371/journal.pone.0046670

**Published:** 2012-10-09

**Authors:** Dongyang Xu, Hong Xu, Ying Ren, Caigang Liu, Xuemei Wang, Hao Zhang, Ping Lu

**Affiliations:** 1 Ultrasound Department, First Hospital of China Medical University, Shenyang, China; 2 Department of Breast Surgery, Tumor Hospital of Liaoning Province, Shenyang, Liaoning Province, People’s Republic of China; 3 Radiology Department, Shengjing Hospital of China Medical University, Shenyang, Liaoning Province, People’s Republic of China; 4 Department of Breast Surgery, General Surgery, First Hospital of China Medical University, Shenyang, Liaoning Province, People’s Republic of China; University of Texas MD Anderson Cancer Center, United States of America

## Abstract

We investigated the expression status of periostin in breast cancer stem cells and its clinical implications in order to lay a foundation for managing breast cancer. CD44+/CD24−/line- tumor cells (CSC) from clinical specimens were sorted using flow cytometry. Periostin expression status was detected in CSC cells and 1,086 breast cancer specimens by Western blot and immunohistochemistry staining, with the CSC ratio determined by immunofluorescence double staining. The relationship between the periostin protein and clinico-pathological parameters and prognosis was subsequently determined. As a result, CSC cells are more likely to generate new tumors in mice and cell microspheres that are deficient in NOD/SCID compared to the control group. Periostin protein was expressed higher in CSC cells compared to the control cells and was found to be related to CSC chemotherapy resistance. Moreover, periostin expression was found to be related to the CSC ratio in 1,086 breast cancer specimens (*P* = 0.001). In total, 334 (30.76%) of the 1,086 breast cases showed high periostin expression. After universal and Spearman regression correlation analysis, periostin was observed to be related to histological grade, CSC ratio, lymph node metastasis, tumor size, and triple-negative breast cancer (all *P*<0.05). Furthermore, periostin was shown to attain a significantly more distant bone metastasis and worse disease-specific survival than those with none or low-expressed periostin protein (*P* = 0.001). In the Cox regression test, periostin protein was detected as an independent prognostic factor (*P* = 0.001). In conclusion, periostin was found to be related to the CSC and an independent prognostic factor for breast cancer. It is also perhaps a potential target to breast cancer.

## Introduction

Breast cancer is the most common cause of death in female malignant tumor disease [1]. In 2008, 1,380,000 new occurrences of breast cancer were diagnosed worldwide, with 458,400 persons dying from breast cancer that same year [2], [3]. Currently, surgical treatment is directed mainly at primary treatment, chemotherapy, radiotherapy, and endocrine therapy, whereas targeted treatment aims to eliminate the residual tumor cells and thus reduce the risk of recurrence and metastasis. Some patients, however, still relapse or metastasize after chemotherapy, radiotherapy, endocrine therapy, and targeted therapy. What causes the poor effects of chemotherapy? Why does targeted treatment have no effect on some patients? These questions remain unanswered.

**Figure 1:**
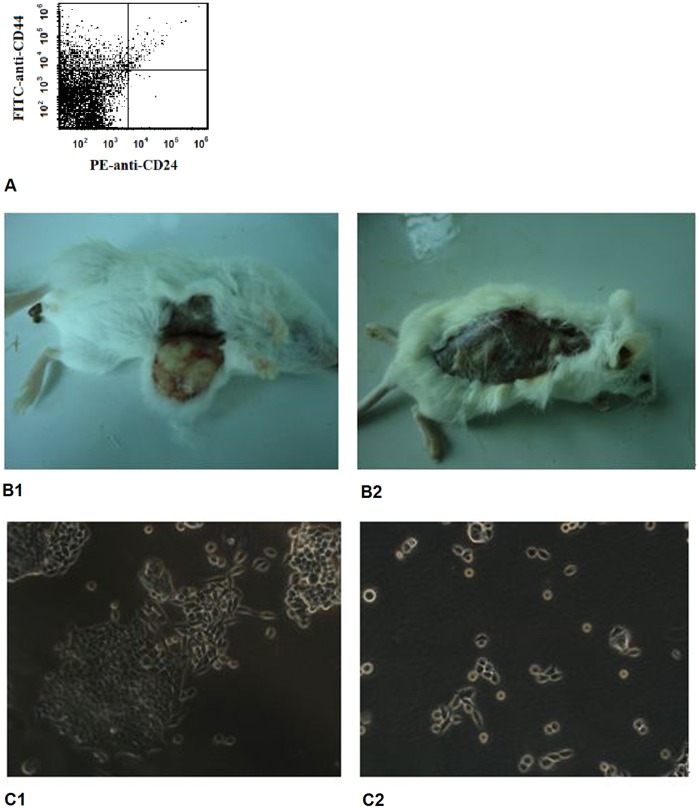
CD44+/CD24- tumor cells got a strong ability to form solid breast tumors and mammospheres. A representative FACS plots to demonstrate the CD44+/CD24- cancer stem cells (A) and a representative tumor in the NOD/SCID mouse showed at the CSC-cell injection site (B1), but not at the non-CSC cell injection site (B2). CSC cells can form mammospheres in the non-adherent culture condition (1-week culture) (C1), whereas no mammosphere was produced in non-CSC cells (C2).

Stem cells, which represent only a very small percentage of the total tumor mass, have been found to be the source of some, and possibly most, cancers [4]. Breast cancer stem cells are a small group of tumor cells with the capacity to self-renew, a strong ability to form solid breast tumors, and the ability to differentiate into a relatively quiescent primitive group of cancer cells that are considered the underlying factor of tumor recurrence an the main reason breast cancers resist therapies [5].

**Figure 2:**
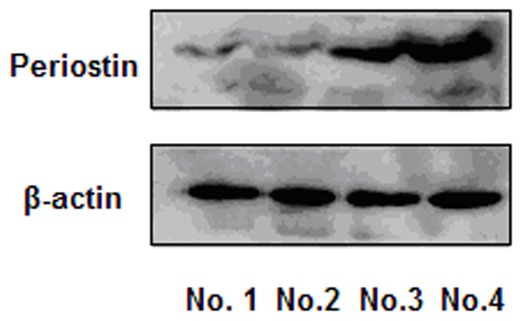
Western blots show that periostin protein was expressed high in CSC cells. Periostin protein was expressed higher in CSC cells (No. 4) compared to the CD44- tumor cells (No. 1), CD44+/CD24+ tumor cells (No. 2), and unsorted cells (No. 3).

Following a better understanding of cancer stem cell theory, stem cell-related genes in malignant tumors have gained more academic attention. Recently, Malanchi et al. found that periostin allows cancer stem cells to maintain, and blocking periostin’s function prevents metastasis [6]. Notably, blocking the periostin protein rarely caused side effects in mice [6]. In another study, Kyutoku et al. [7] reported that periostin plays a pivotal role in how breast cancer progresses and metastasizes. Administering the periostin antibody prolonged cell survival by inhibiting the progression and metastasis of 4T1 cells; therefore, developing the periostin antibody further, including generating a humanized antibody, may provide a new therapeutic agent against breast cancer.

Periostin is a kind of bone adhesion molecule that regulates osteoblast adhesion and differentiation and is classified among the extracellular matrix (ECM) proteins [8]. It presents as part of the extracellular matrix in natural conditions and plays an important role in fetal development. In adults, only some specific organs such as the breast, bone, skin, and intestines have periostin activity [9]. Periostin is reported to be involved in tumor EMT, extracellular matrix degradation, tumor invasion, and distant metastasis, but the mechanism by which it operates is still unclear [10], [11]. Currently, the periostin expression status in breast cancer stem cells (CSC) and clinical implications for breast cancer are also unclear. In the present study, we try to sort and identify breast cancer stem cells investigate the expression status of periostin in those cells cells, and evaluate the clinical implications of periostin in breast cancer. Gaining this knowledge will lay a foundation for managing breast cancer.

## Materials and Methods

### Patients and Tissue Specimens

A total of 1,086 patients who had histologically confirmed breast cancer and who underwent radical operations in the Tumor Hospital of Liaoning province and China Medical University between January 2001 and January 2006 were enrolled for immunohistochemical and immunofluorescence double staining and prognostic analysis. The mean age was 50.73±10.28 years (range from 27 to 80 years). The criteria to include a patient in this study were as follows: (1) curative operations were performed; (2) resected specimens were pathologically examined; (3) more than 10 lymph nodes were pathologically examined after the operation; and, (4) a complete medical record including the ER, PR, Her2, p53, and Ki67 status was available. The study protocol was approved by the Ethics Committee of China Medical University and Liaoning Tumor Hospital and a written informed consent was obtained from all participants involved in the study. The animal experiments were also approved by the Ethics Committee of China Medical University.

**Figure 3:**
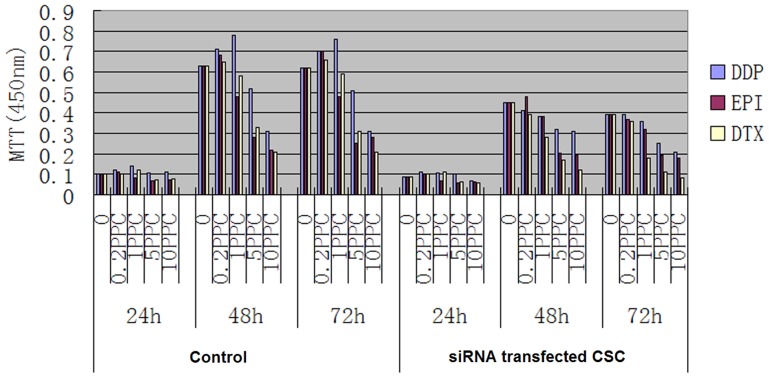
MTT assay shows that the cells exposed to periostin siRNA showed a significant decrease in IC50 among the DDP, EPI, and DTX compared with the control siRNA group (control group) (*P*<0.01).

### Identifying the Ability of CSC Cells to Form Tumors

The clinical specimens were digested into single tumor cells using collagenase III. The tumor cells were suspended in 100 µl/10^6^ cells of HBSS with 2% HICS. The samples were then washed twice with HBSS/2% HICS and suspended. Antibodies, including anti-CD2, -CD3 -CD10, -CD16, -CD18, -CD31, and anti-CD326 were added and incubated for 20 min on ice and then washed twice with HBSS/2% HICS. Lineage+ cells were first eliminated using anti-CD2, -CD3 -CD10, -CD16, -CD18, -CD31, and anti-CD326 during flow cytometry. Dead cells were eliminated using the viability dye 7AAD. Next, CD44+/CD24- tumor cells were sorted by CD44 and CD24 in flow cytometry. The selected cells to be injected were then suspended in 1640/Matrigel mix (1∶1 volume) and injected into the appropriate area of the mammary fat pad. The tumorigenicity experiments were repeated three times.

### Mammosphere Generation Test

For this step, Complete MammoCult™ Medium (Human) was prepared by adding 50 mL of thawed MammoCult™ Proliferation Supplements (Human) to 450 mL of MammoCult™ Basal Medium (Human). Single cells were plated on ultralow attachment plates (Corning, Acton, MA, USA) at a density of 20,000 viable cells/mL in Complete MammoCult™ Medium. The number of spheres for each well was evaluated after 7 d of culture.

### siRNA Transfection and the Effect Confirmation

Periostin siRNA and control siRNA were designed and synthesized by Santa Cruz Biotechnology, Inc. Transfection was performed in 50 to 60% confluent cells using Lipofectamine 2000 Reagent (Invitrogen, Carlsbad, CA, USA) according to the manufacture’s protocol. Briefly, siRNA and Lipfectamin 2000 in Opti-MEM I Reduced Serum Medium (Invitrogen, Carlsbad, CA, USA) were diluted separately and mixed gently. After incubation for 15 min at room temperature, they were combined and incubated for another 15 min at room temperature. Next, the complexes were added to the culture plates or wells with cells and the medium without serum, and then the final concentration of siRNA to 25 nM was made. After incubating the cells at 37°C in a humidified CO_2_ incubator for 6 h, the medium was replaced with complete medium. Transfecting only with Lipofectamine 2000 acted as a mock transfection. The downregulation of periostin by siRNA was confirmed by RT-PCR and Western blot at the indicated time points.

**Figure 4:**
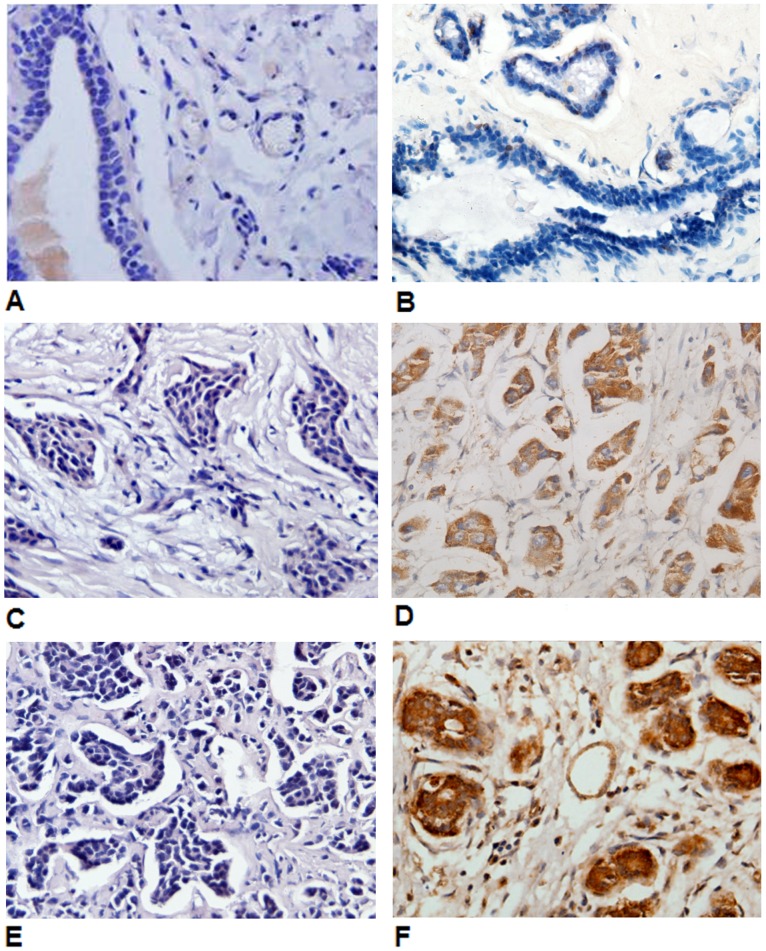
Periostin was located in the cytoplasm and membrane of the breast cancers. It was observed that Periostin was either not expressed or expressed low in paracancerous tissues (A, x400) and atypical hyperplasia tissues (B, x400); expressed low in cases without lymph node metastasis (C, x400) and expressed high in cases with lymph node metastasis (D, x400); expressed low in the non-triple-negative breast cancers (E, x400) and expressed high in triple-negative breast cancers (F, x400).

### Treatments with Chemotherapeutic Agents and Measuring Cell Viability

When the above cells cultured as monolayers were healthy and were 80 to 90% confluent, they were washed with warm Hank’s Balanced Salt Solution (HBSS). The cells were scraped gently from the dish using a sterile cell scrape. The scraped cells were then suspended in Complete MammoCult medium and counted. The sensitivity of the cells to three chemotherapeutic drugs were examined using the Cell Counting Kit-8 (CCK-8) technique. Cells were plated at a density of 5×104/mL cells per well into Ultra-Low Adherent 96-well plates containing 100 µl Complete MammoCult medium and treated with for concentration of DDP (2.5 µg/ml/PPC (plasma peak concentration)), EPI (0.78 µg/ml/PPC), and DTX (3.7 µg/ml/PPC) as follows: 0.2, 1.0, 5.0, 10.0 PPC. CCK-8 reagent was added to each well and incubated for 2 h before reading at wavelength of 450 nm. The cells were counted at 24, 48, 72 and 96 h with CCK-8.

**Figure 5:**
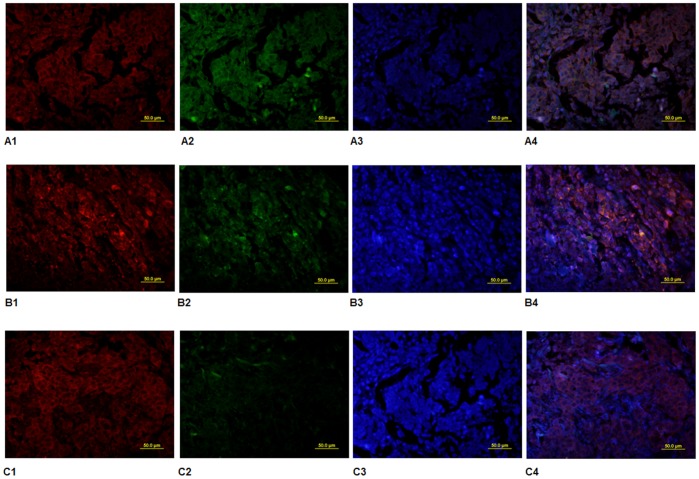
The CSC ratios in 1,086 cases were observed. Images show <5% (A), 5 to 10% (B), and >10% (C) CSC ratios in the specimens. Staining was performed with: mouse anti-human CD44 antibody and PE conjugated secondary (1); rabbit anti-human CD24 antibody with FITC conjugated secondary (2); and DAPI to detect nuclei (3). An overlay of panels (1) and (2) is presented in (4).

### Western Blot Analysis

For Western blot analysis, cells were lysed with the buffer [0.1% SDS, 50 mmol/L Tris-HCl.(PH7.6), 1% NP-40, 150 mmol/L NaCl, 2 mg/ml aprotinin, 2 mg/ml leupeptin and 7 mg/ml PMSF]. The protein concentrations were determined using the BCA Protein Assay kit (Pierce Biotechnology, Inc., Rockford, IL, USA). Thirty micrograms of protein were separated on 10% SDS-PAGE gels and transferred to a PVDF membrane. After blocking, the membrane was incubated with anti-periostin antibody (1∶500, Biorbyt Ltd., United Kingdom) at 4°C overnight. After washing, the membrane was incubated with a secondary antibody at a dilution 1∶2,000 at room temperature for 1 h. Proteins were detected with the ECL Kit (Varsal Instruments, Beijing, China), and anti-β-actin antibody (Sigma-Aldrich, St. Louis, MO, USA) was used as a loading control. Densitometry was performed by Gel-pro Analyzeftware (Media Cybernetics, Silver Spring, MD, USA).

### Immunohistochemistry Experimental Procedures

Thin slices of tumor tissue for all cases received in our histopathology unit were fixed in 4% formaldehyde solution (pH 7.0) for periods not exceeding 24 h. The tissues were processed routinely for paraffin embedding, and 4 µm-thick sections were cut and placed on glass slides coated with 3-aminopropyl triethoxysilane for immunohistochemistry. Tissue samples were stained with hematoxylin and eosin, and the pathologist Chen B and Wang MX determined the histological type and tumor grade.

**Table 1:** Correlations between Periostin expression and clinico-pathological features(n = 1086).

Variables	n	Periostin^–^	Periostin ^+^	X^2^ value	*P* value
Age					
<40 Y	174	110	64	3.534	0.073
>40 Y	912	642	270		
Tumor size					
T1	173	104	69	20.665	0.001
T2	836	580	256		
T3	69	62	7		
T4	8	6	2		
Histological grade				11.576	0.003
I	84	63	21		
II	732	524	208		
III	270	165	105		
Histological type				2.477	0.290
Ductal	831	582	249		
Lobular	96	66	30		
Others	159	104	55		
HER2 status				0.391	0.218
negative	814	558	256		
positive	272	194	78		
Metastatic nodes				34.133	0.001
negative	467	437	130		
positive	519	315	204		
Distant metastasis				185.466	0.001
negative	798	644	154		
positive	288	108	180		
Triple-negative breast cancer				39.052	0.001
Yes	295	162	133		
No	781	590	201		
CSC ratio				536.135	0.001
<5%	621	603	18		
5–10%	364	128	236		
>10%	101	21	80		

Briefly, immunohistochemical staining was performed using the standard streptavidin-peroxidase (SP) method with the UltraSensitive TM S-P Kit (Maixin-Bio, China) according to the manufacturer's instructions, and signals were visualized using the DAB substrate, which stains the target protein yellow. Briefly, one paraffin-embedded block of the tissue was cut at 4 µm and placed on poly-L-lysine coated slides. The slides were deparaffinized in xylene, rehydrated in a gradient of ethanol solutions, and then immersed in 10 mM sodium citrate buffer (pH 6.0), pretreated in a microwave oven for 10 min, followed by a 10-minute rinse with phosphate-buffered saline (PBS). The sections were incubated with 3% hydrogen peroxide for 10 min to block endogenous peroxidase activity at room temperature. Nonspecific reactions were blocked by incubating the sections in a solution containing normal serum. Then the slides were incubated in a humid chamber at 4°C overnight with primary antibody. Following washings with PBS, sections were incubated for 30 min in the secondary biotinylated antibody (Multilink Swine anti-goat/mouse/rabbit immunoglobulin; Dako, Inc.). Following washings, Avidin Biotin Complex (1∶1000 dilution, Vector Laboratories, Ltd.) was applied to the sections for 30 to 60 min at room temperature. The immunoreactive products were visualized by catalysis of 3,3′-diaminobenzidine (DAB) by horseradish peroxidase in the presence of H_2_O_2_ following extensive washings. Sections were then counterstained in Gill’s Hematoxylin and dehydrated in ascending grades of methanol before clearing in xylene, then mounting under a coverslip.

**Table 2:** Spearman correlation analysis between clinic-pathological features and Periostin.

Clinic-pathological features	Periostin expression (p; Spearman correlation)
age	0.060(0.057)
Histological grade	0.001(0.100)
Histological type	0.460(0.001)
HER2 status	0.075(0.036)
CSC ratio	0.001(0.170)
Lymph node metastasis	0.001(0.177)
Tumor size	0.001(0.118)
Triple-negative breast cancer	0.001(0.190)

**Table 3:** Multivariate analysis of the factors related to post-operative distant metastasis.

Characteristic	Exp(B)	95% CI for Exp(B)	*P* value
age	0.335	0.211–0.532	0.001
Histological grade	3.574	2.540–5.028	0.001
Histological type	1.208	0.755–1.824	0.125
CSC ratio	1.193	0.845–1.685	0.316
Lymph node metastasis	4.836	3.239–7.219	0.001
Tumor size	1.475	1.029–2.114	0.035
Triple-negative breast cancer	8.434	5.701–12.476	0.001
Periostin	5.829	3.582–9.485	0.001
Constant	0.005		

CI = confidence interval.

To score periostin as immuno-positive staining, the positive cells appeared as a yellow to brown color in the nucleus and/or cytoplasm. Periostin expression was classified semi-quantitatively according to the following criteria: 0 if <1% of neoplastic cells discretely expressed periostin; 1+ if ≥1 and <10% of morphologically unequivocal neoplastic cells discretely expressed periostin; and, 2+ if ≥10% of morphologically unequivocal neoplastic cells discretely expressed periostin. Samples scored as 1+ or 2+ were considered positive. Nuclear staining for ER, PR, and P53 was graded 1+ if <10% of the cells were stained, 2+ if 10–50% of the cells were stained, and 3+ if >50% of the cells were stained. Grades 2+ and 3+ were considered positive, whereas absence of staining and 1+ staining were considered negative. Similar standards were used for staining intensity in HER-2 / neu; only grade 3+ (high intensity) was considered positive.

### Double Immunofluorescence Staining

Surgical resected breast cancer tissues were fixed and cut at a thickness of 4 µm. The specimens were stained with control IgG, mouse anti-human CD44 (1∶50 dilution, Becton, Dickinson and Company, USA), rabbit anti-human CD24 polyclonal antibodies (1∶50 dilution, Becton, Dickinson and Company, USA) at 4°C for 12 h. After being washed, the cells were incubated with biotinylated goat anti-mouse IgG secondary antibodies (1∶1,000 dilution) in PBS for 30 min at room temperature, and the bound antibodies were detected with PE and FITC, followed by mounting with DAPI medium (Vector Labs, Burlingame, VT, USA). The expression of CD44 and CD24 was examined under a fluorescence microscope. The cell staining was observed under a fluorescence microscopy. We randomly selected five regions under the fluorescence microscopy and the CSC ratio was the mean of the five ratios in the selected areas.

**Table 4:** Correlations between periostin expression and distant metastasis(n(%)).

Organs metastasis	n	Periostin-	Periostin ^+^
Bone	134	42(38.89)	92(51.11)
Lung	59	22(20.37)	37(20.56)
Liver	46	20(18.52)	26(14.44)
Ovarian	22	8(7.41)	14(7.78)
Others	27	16(14.81)	11(6.11)

### Statistical Analysis

All data were analyzed with SPSS Statistics software (Version 13.0, Chicago, IL, USA). Relationships between periostin and other parameters were studied using the chi-square test, Fisher’s extract test, or independent *t* tests. Disease-specific survival was analyzed using the Kaplan-Meier method. The log-rank test was used to analyze survival differences. Multivariate analysis was performed using the Cox proportional hazards model selected in forward stepwise. A *P* value of less than 0.05 was considered statistically significant.

## Results

### Identifying the Stemness of CSC Cells

We found that the mean percentage of CD44^+^CD24^-^ cells in total tumor cells was from 3.75% to 33.11% (representative samples in Fig. 1A). A quantity of 10^3^, 10^4^, 10^5^, and 10^6^ CSC cells that were separated from the solid tumors were separately injected into the right mammary fat pad of four SCID mouse. Meanwhile, the same number of non-CSC cells was injected into the left mammary fat pad as a control. Eight weeks post-injection, 10^3^ CSC cells successfully formed a tumor (2/4), while non-CD44+/CD24- tumor cells failed to form tumors until attaining 10^6^ cells (1/4) (P<0.05, Fisher’s extract test, Fig. 1B1 and B2).

After 7 days of culture, single-cell suspensions of CSC cells that were separated from the solid tumors produced viable mammospheres (20–100 µm), which could be passaged further. No mammosphere was produced by the non-CSC cells in the same culture condition (see Fig. 1C1 and C2).

CSC cells generated new tumors in mice and cell microspheres that were deficient in NOD/SCID compared to the control group. Furthermore, periostin protein was expressed higher in CSC cells compared to the control cells (Figure 2). Moreover, periostin expression was found to be related to the CSC ratio in 1,086 breast cancer specimens (*P* = 0.001).

**Table 5:** Correlations between periostin expression and chemotherapeutic resistance in breast cancers (n = 135; n(%)).

Chemosensitivity	n	Periostin^–^	Periostin ^+^	X^2^ value	*P* value
CR	15	11	4(26.67)	8.017	0.046
PR	60	43	17(28.33)		
SD	32	18	14(43.75)		
PD	28	12	16(57.14)		

CR: complete response; PR: partial response; SD: stable disease; PD: progressive disease.

### Expression of Periostin was Blocked Efficiently by RNAi

By replacing the serum culture medium at 6 h after siRNA transfection, we observed that the successfully transfected CSC cells had green fluorescent when examined using fluorescence microscopy. The positive rate of transfected cells was 66.12±18.46% in the CSC cells. The expression of periostin was examined by RT-PCR and Western blot at 48 h after siRNA transfection. The efficiency reached more than 82% at the protein level in the CSC cells.

### Periostin Downregulation Sensitizes to Chemotherapy Drugs in CSC Cells

To investigate whether downregulation of periostin expression has the potential to sensitize CSC cells to chemotherapy, a combination treatment of periostin-specific siRNA with chemotherapy drugs was performed. Twenty-four hours after transfection with siRNA, cells were treated with DDP, EPI, and DTX at 0, 0.2 PPC, 1 PPC, 5 PPC and 10 PPC scaled concentrations for 72 h. The IC50 was determined by MTT assay. Figure 3 shows that the cells exposed to periostin siRNA showed a significant decrease in IC50 among the three drugs when compared with the control siRNA or no treatment (*P*<0.01).

**Figure 6:**
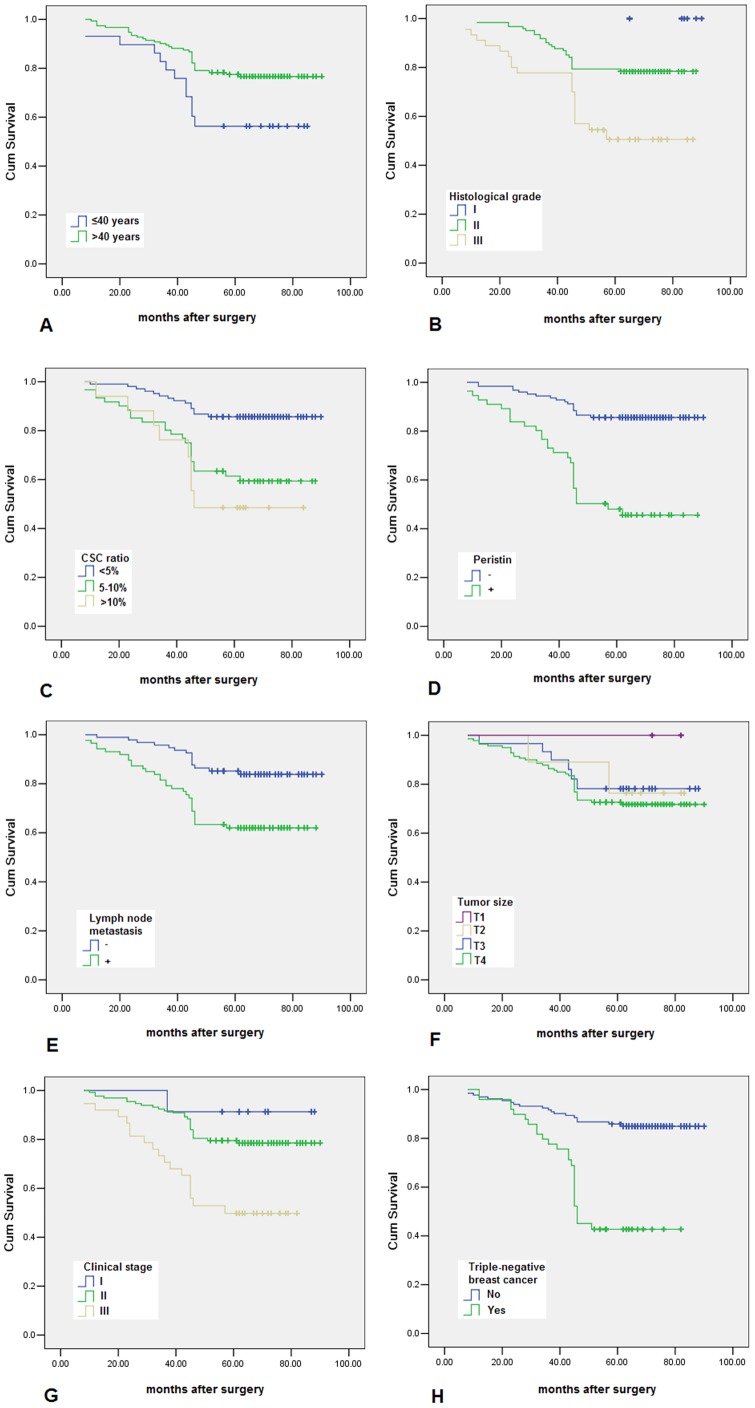
Periostin, along with age, histological type, lymph node metastasis, and triple-negative breast cancer were shown to be related to the prognosis (*P* = 0.001, 0.002, 0.001, 0.004, and 0.001, respectively).

**Table 6:** Cox model regression analysis of the breast cancer prognostic factors.

Varies	OR	95% CI for OR	*P* value
age	1.410	1.133–1.755	0.002
Histological grade	1.545	1.355–1.762	0.001
Lymph node metastasis	1.245	1.074–1.443	0.004
Triple-negative breast cancer	2.597	2.121–3.179	0.001

### Periostin Expression in Breast Cancer and its Relationship with Clinicopathological Characteristics

Immunohistochemical examination showed that periostin was located in the cytoplasm and membrane of the breast cancers (Figure 4). It was also observed that periostin protein was expressed significantly higher in breast cancer tissues compared to paracancerous tissue and atypical hyperplasia tissues (30.76% vs 7.92% vs 5.99%, respectively) (Fig. 4A and 4B). The cases with high periostin expression intended to develop into lymph node and postoperative distant metastasis (*P* = 0.001 and 0.001, repsectively) (Fig. 4C and 4D). Moreover, 133 (45.08%) of the 295 triple-negative breast cancers showed periostin expression compared to 201 (25.74%) of the 781 cases of non-triple-negative breast cancers (*P* = 0.001) (Fig. 4E and F).

We also determined the CSC ratio in the 1,086 patients. CSC ratios in 621 (57.18%) cases were <5%; 364 (33.52%) were 5 to 10%; and 101 (9.3%) were >10% (Table 1, Fig. 5). In total, 334 (30.76%) of the 1,086 breast cancer cases showed high periostin expression. After universal analysis, periostin was observed to be related to tumor size, histological grade, lymph node metastasis, postoperative distant metastasis, triple-negative breast cancer, and CSC ratio (*P* = 0.01, 0.003, 0.001, 0.001, 0.001, and 0.001 respectively) (Table 1).

Spearman correlation regression analysis showed that periostin expression has a linear correlation to histological grade, CSC ratio, lymph node metastasis, tumor size and triple-negative breast cancer (*P* = 0.001, 0.001, 0.001, 0.001, and 0.001 respectively) (Table 2). After multivariate analysis, age, histological grade, lymph node metastasis, tumor size, triple-negative breast cancer, and periostin expression were related to post-operative distant metastasis (*P* = 0.01, 0.001, 0.001, 0.035, 0.001, and 0.001, respectively) (Table 3).

### Postoperative Recurrence Pattern and Chemotherapeutic Resistance

In the present study, the patients with positive periostin expression attain a significantly more distant metastasis rate. Of the 334 cases with high periostin expression, 180 (53.89%) developed 5-year postoperative distant metastasis, whereas only 12.68% of patients without periostin expression developed 5-year postoperative distant metastasis (*P* = 0.001). After subgroup analysis, a significant association between periostin expression and postoperative bone metastasis (Table 4) was observed.

We further studied the relationship among age, tumor size, histological grade, histological type, molecular type, CSC ratio, periostin expression and chemotherapeutic sensitivity in 135 neoadjuvant chemotherapy breast cancers. Finally, molecular type, CSC ratio and periostin expression were observed significantly related to the chemosensitivity (P = 0.01, 0.012 and 0.046 respectively). Periostin expression was expressed in 26.67%, 28.33%, 43.75%, and 57.14% in complete response (CR), partial response (PR), stable disease (SD), and progressive disease (PD) patients, respectively (Table 5).

### Prognostic Analysis

In the prognostic analysis, patients with cancer expressing periostin, along with age, histological grade, lymph node metastasis, and triple-negative breast cancer, were shown to attain a poorer disease-specific survival than those with no or low expressed periostin protein (*P* = 0.001, 0.002, 0.001, 0.004, and 0.001, respectively) (Figure 6). In the Cox regression test, periostin protein was detected as an independent prognostic factor (*P* = 0.001) (Table 6).

## Discussion

Periostin is a 93.3-kD secreted cell adhesion protein, which has been shown to be a critical regulator of bone metabolism, hypertensive nephropathy, and wound repair [12], [13]. Recent studies have revealed that periostin plays an important role in tumor epithelial-mesenchymal transition, tumor angiogenesis, and tumor development. It is upregulated in a wide variety of cancers including colon, pancreatic, ovarian, head, and gastric cancer [14], [15].

Recently, Malanchi et al demonstrated that stromal periostin is crucial for metastatic colonization by regulating the interactions between cancer stem cells and their metastatic niche [6]. Periostin mediates the crosstalk between cancer stem cells and their niche. It is considered required to maintain cancer stem cells, and blocking its function prevents metastasis. Moreover, blocking the periostin protein rarely caused side effects in mice. Hence, periostin may be a potential breast cancer treatment target. Currently, the expression status of periostin protein in breast cancer and its relationship to the biological behavior of the disease are still unclear [16]. Furthermore, studies that have addressed the relationship between periostin and chemotherapy sensitivity and prognosis of breast cancer are still sparse [17].

Tumor stem cells have been found to be the source of most cancers and the culprit of tumor recurrence and metastasis and drug resistance [18]. In recent studies, periostin was reported as a bridge between cancer stem cell and metastasis [6], [8]. No studies to date, however, have examined the relationship among periostin expression status and breast cancer CSC ratio, chemotherapy sensitivity, and the clinical implications of breast cancer. In the current study, we sorted and identified the breast cancer CSC from clinical specimens, observing that periostin was expressed high in breast cancer CSC compared to the control group. Moreover, drug sensitivity tests showed that a combination treatment of periostin-specific siRNA with chemotherapy drugs could significantly increase the apoptosis of breast cancer CSC. The outcome demonstrated that periostin plays an important role in breast cancer’s resistance to chemotherapy.

We also investigated the relationship between periostin expression and the biological behavior of breast cancer stem cells and the clinicopathological characteristics of breast cancer. Periostin protein was observed to be expressed significantly higher in cancerous tissues than adjacent-tumor tissues. Moreover, periostin protein was found to be related to tumor size, histological grade, lymph node metastasis, postoperative distant metastasis, triple-negative breast cancer, and CSC ratio in the 1, 086 breast cancers studied. We also studied the relationship between periostin expression and chemotherapeutic sensitivity. We found that periostin expression was significantly related to a poor chemotherapy response in breast neoadjuvant chemotherapy.

After survival analysis, periostin was shown to attain a significantly more distant postoperative bone metastasis and attained significantly poorer postoperative disease-specific survival. Indeed, the Cox regression test showed periostin protein was detected as an independent prognostic factor. These outcomes suggest that periostin is associated with breast cancer CSC, suggesting that its expression may be implicated in self-renewal and tumorigenesis by activating its downstream target genes. Periostin may also play a role in breast cancer oncogenesis and may be a potential biomarker for metastasis and chemotherapy resistance of breast cancer.

### Conclusion

The present study found that periostin was highly expressed in CSC cells and could be a potential biomarker for the bone metastasis and chemotherapy resistance of breast cancer tumors. The underlying genetic mechanism of periostin regulating the breast cancer CSC is still unclear, however, and needs further investigation.
